# Supplementary material for: A UMLS-based spell checker for natural language processing in vaccine safety
Source: BMC Med Inform Decis Mak. 2007 Feb 12;7:3. doi: 10.1186/1472-6947-7-3 (PMC1805499; doi:10.1186/1472-6947-7-3)
Supplement: Additional file 13 — RAP application directory. Sets up directory for RAP (RDF application for PHP) source code [file 1472-6947-7-3-S13.gz › rap/doc/phpdoc/model/_infModel_InfModelP_php.html]

xml version="1.0" encoding="iso-8859-1"?


Docs for page InfModelP.php


## /infModel/InfModelP.php

Description

Description |
Includes

Includes

Description |
Includes

require\_once
(RDFAPI\_INCLUDE\_DIR.'infmodel/InfRule.php')
(line 16)

require\_once
(RDFAPI\_INCLUDE\_DIR.'infmodel/InfStatement.php')
(line 17)

require\_once
(RDFAPI\_INCLUDE\_DIR.'infmodel/InfModel.php')
(line 18)

require\_once
(RDFAPI\_INCLUDE\_DIR.'infmodel/InfModelB.php')
(line 19)

require\_once
(RDFAPI\_INCLUDE\_DIR.'infmodel/InfModelF.php')
(line 20)

Documentation generated on Wed, 20 Oct 2004 13:59:17 +0200 by phpDocumentor 1.3.0RC3
